# The TRICK-TIP Rhinoplasty: Tip of the Nose Preservation Using the Combined Synergy of Open and Closed Approaches

**DOI:** 10.1007/s00266-024-03901-w

**Published:** 2024-03-14

**Authors:** Francisco Villegas-Alzate

**Affiliations:** 1 Private practice, Clínica San Francisco, Tuluá, Colombia; 2 Universidad del Valle, Cali, Colombia; 3 Unidad Central del Valle, Tuluá, Colombia

**Keywords:** Rhinoplasty/methods, TRICK-TIP, Patient satisfaction, Nose esthetic techniques, Patient reported outcome measures, Nose deformities acquired

## Abstract

**Background:**

This study introduces and assesses the outcomes of a novel rhinoplasty technique, TRICK-TIP (Transcolumellar and Inter Cartilaginous Keystoning with Tip preservation), employing a combined open and closed approach with tip anatomy conservation and structured tip support.

**Methods:**

The procedure involves a low stairstep columellar sectioning, followed by transmembranous and intercartilaginous incisions without skin dissection in the columella or tip. Elevating the entire mobile nose as a three-layered flap provides extensive access to the entire nasal pyramid and septum. Tip modifications, including retrograde cephalic cartilage resection and supratip skin thinning, are performed based on individual cases. A key columellar strut is frequently used, initially sutured in the interdomal space and then turned down for height adjustment and final fixation. Interdomal sutures, supratip sutures, and alar resection are implemented as needed.

**Results:**

One hundred twenty patients participated, with high satisfaction and a low frequency of adverse effects reported using four FACE-Q™ questionnaires. One hundred and two independent raters evaluated pre and postoperative photographs, scoring “overall nose result” as 3.6 out of 5, with minimal or absent nostril deformities (1.84), soft triangle deformities (1.73), and columellar external scar deformity/visibility (1.35) where 1 is the absence of the deformity and 5 is disfigurement. Complications were absent, and revisions were infrequent.

**Conclusions:**

The combined benefits of the wide-open approach, shortened surgery duration, and nasal tip preservation contribute to outcome optimization. TRICK-TIP rhinoplasty is characterized by simplicity, enabling targeted modifications, preventing soft triangle and rim complications, and facilitating essential tip support while maintaining favorable results.

*Level of Evidence V* This journal requires that authors assign a level of evidence to each article. For a full description of these Evidence-Based Medicine ratings, please refer to the Table of Contents or the online Instructions to Authors www.springer.com/00266.

## Introduction

Gillies, in 1920, was credited with using the transcolumellar incision (“elephant trunk”). However, the allegory was not found in reading his book [1]; this term may have been used in his lectures, referring to the incision and, probably, to the tubed forehead flap. Other rhinoplasty authors have used this metaphor occasionally [2].

After utilizing intercartilaginous incisions for "Reductive Rhinoplasty" in 1921, Rhéti found the closed approach, insufficient to modify alar cartilages. Subsequently, he devised a supplementary method, featuring a transverse columellar high incision connected to marginal incisions, elevating the skin to expose the alar cartilages. He confined the dissection to the tip, deeming it inadequate for dorsal modifications [3].

Later, Sercer citing Rhéti, extended the approach to the entire nose using the term “Decortication Rhinoplasty” [4]. Padovan demonstrated its usefulness as it is used today [5]. Goodman popularized the incision in the USA as the “Butterfly Incision” [6], after a while, “Open Rhinoplasty” was the most cited term and became widely embraced for its advantages [7].

The open rhinoplasty enhances visualization for precise modifications, addressing complex deformities. Drawbacks include the need for specialized surgical skills, prolonged surgical time, meticulous dissection, and increased costs [8]. Additional dissection in the vestibular skin may be necessary to prevent retractions and notching, posing risks of irregularities, asymmetries, and indentations of the alar rim and soft triangles [9, 10]. Skin necrosis, particularly in secondary cases, may be associated [11, 12].

On the other hand, closed rhinoplasty offers several advantages, including simplicity, shorter surgical time, no visible external scarring, reduced swelling, faster recovery, and relative preservation of the columella and nasal tip cartilages with minimal disruption to the blood supply. Nevertheless, it has drawbacks, primarily limited visibility and less precise modifications, making it less suitable for addressing severe nasal deformities [13]

Rhéti transformed classical endonasal surgery into a dual approach by modifying alar cartilages through additional transcolumellar and marginal incisions [14]. Currently, surgeons make modifications to closed rhinoplasty, extending incisions to the alar creases and nasal sill to utilize advantages and mitigate disadvantages of both approaches [15–24].

The "TRICK-TIP" technique, an acronym for "Transcolumellar and Inter-Cartilaginous Keystoning with Tip Preservation," involves elevating the nasal tip as a three-layered chondro-cutaneous flap for a broad surgical field. Enhanced visibility allows precise modifications while preserving anatomical tip relationships, avoiding dissection between the skin and tip cartilages. By circumventing such dissection, the technique aims for tip preservation, simplicity and satisfactory results. To our knowledge, the TRICK-TIP technique is unique and not previously published, demonstrating easiness, favorable outcomes, and reduced complications.

### Methods

A single surgeon's retrospective experience with aesthetic TRICK-TIP rhinoplasty from 2010 to 2022 is examined.

Inclusion criteria encompassed consecutive primary or secondary aesthetic cases with various deformities and complaints, featuring follow-up periods longer than 12 weeks and assessable postoperative photographs. Patients were informed about the procedure, including potential risks and benefits, with assurances of confidentiality for academic research, education, or scientific publications using their facial photographs. Excluded were cases for reconstructive purposes. All patients underwent presurgical consultations and had surgeries in an academic practice within a multispecialty private hospital under general anesthesia.

### Surgical Technique

**Fig. 1:**
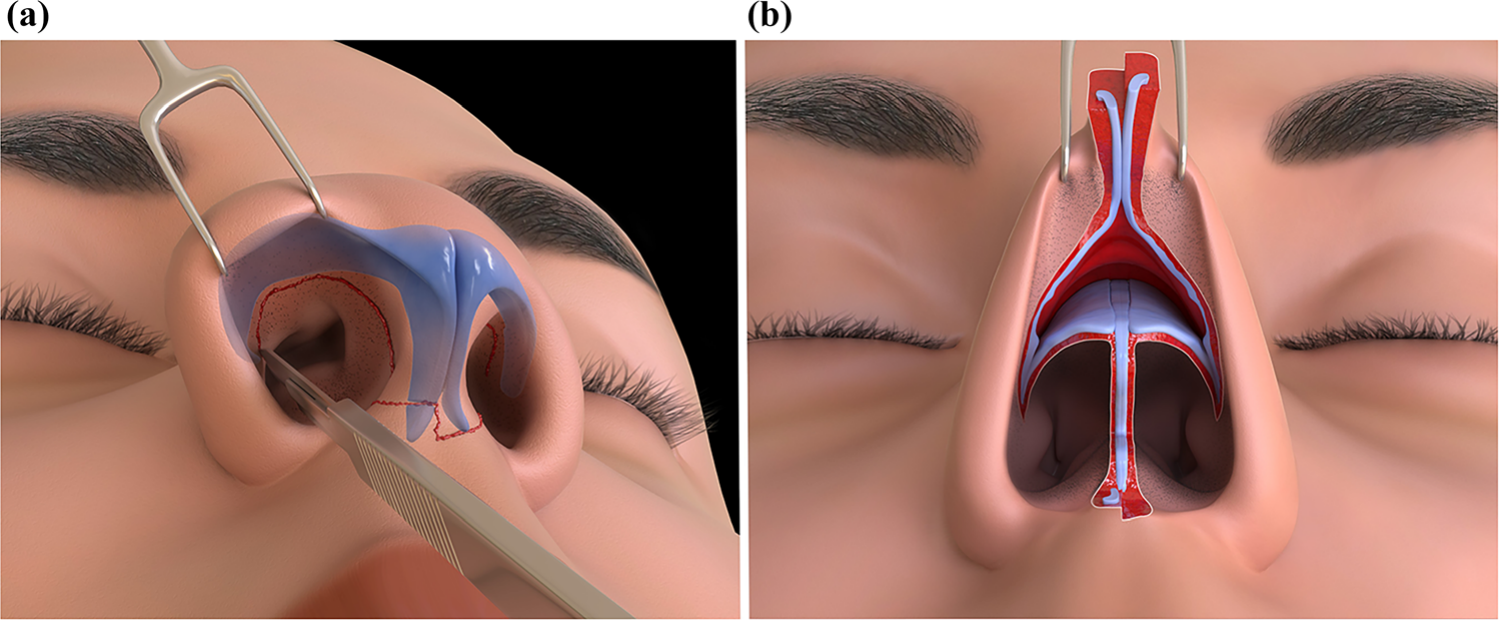
TRICK-TIP rhinoplasty (Transcolumellar and Inter Cartilaginous Keystoning with Tip preservation). **a** Depiction of intercartilaginous and transcolumellar incisions. Note the low positioning of the stairstep incision near the labio-columellar crease. Sectioning of one or both crural plates is typical. **b** The entire mobile tip and columella are elevated as a composite flap, allowing for easy access to the nasal pyramid

A stairstep incision is made in the lower third of the columella, near the crural foot plates and just above the labio-columellar crease. This incision progresses in-depth, involving sectioning of one or both crural feet, and connects to a vertical transfixing incision through the membranous septum behind the medial cruras, extending up to the nasal vestibular apex. Bilateral inter-cartilaginous incisions follow. The mobile nose can then be elevated without additional dissecting maneuvers. The elevated mobile nose (tip and columella) pivots at the septal angle, where the dissection to the dorsum continues up to the nasion and can be extended laterally on the nasal sidewalls (Fig. 1).

A supra-perichondral level of dissection is preferred in the cartilaginous vault to maintain upper lateral cartilages integrity, preventing ruptures during folding maneuvers in chondroplastic techniques with auto-spreaders or spreader flaps and spreader grafts that involve plication and suturing [25].

**Fig. 2:**
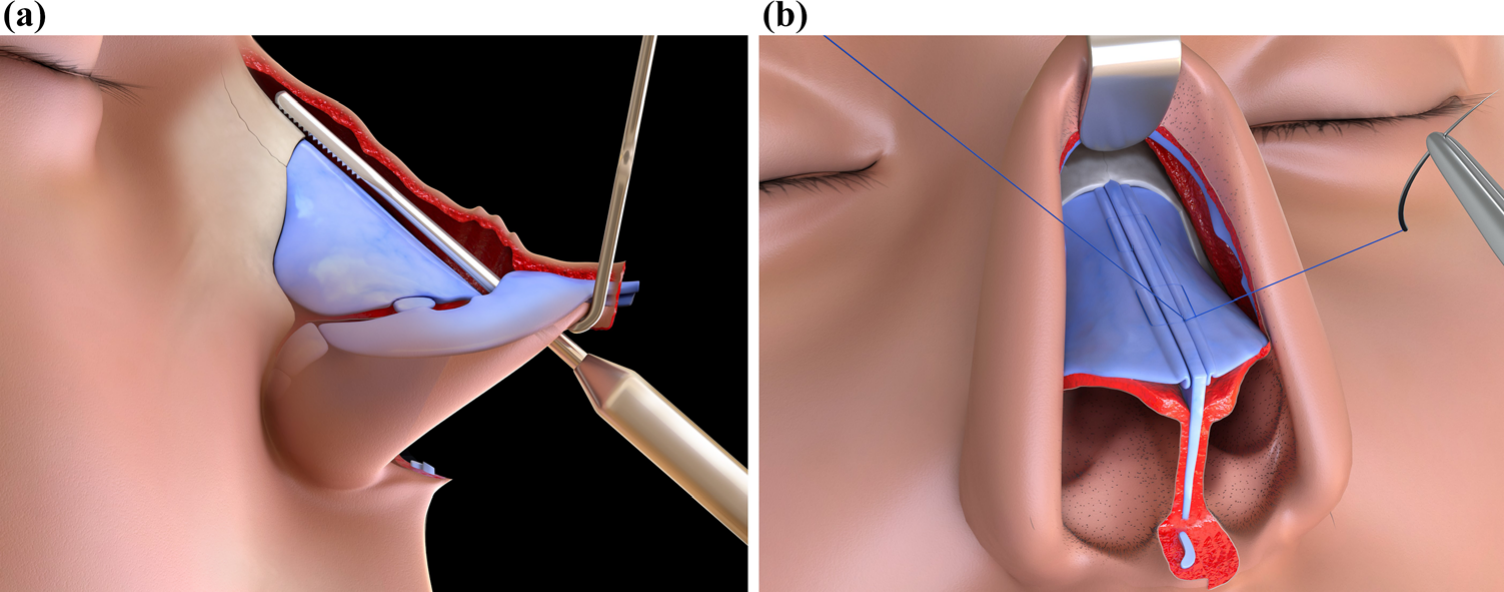
TRICK-TIP rhinoplasty Enhances Access. **a** After the dorsum and skin are separated, the bony vault can be reformed. **b** The cartilaginous vault can be molded with improved visibility

In the bony nasal vault, subperiosteal dissection starts laterally using sharp dissectors. It progresses from lateral to medial and proximal to distal, creating an optical cavity from the caudal septum to the nasion. Efforts are made to preserve periosteum integrity whenever possible. Septoplasty and septal cartilage graft harvest can be performed as needed, either through the caudal septum or from above if the upper lateral cartilages are surgically separated from the dorsum (Fig. 2).

Tip modifications are completed from underneath, with retrograde cartilage trimming in the lateral crura or domal areas as needed. In most cases, minimal cartilage reversing dissection is employed to spare the skin from resection. Modifications to the interdomal tissues, ligaments, fat, and supratip areas can be achieved through direct excision. Fat removal can be completed by using curettage with a 2-mm Frazier’s suction cannula. To address supratip dead space, a suture unites the skin to the cartilaginous vault, reinforcing stability between the dorsum and tip. Sutures can either be immediately tied (Guyuron) [26] or left for tying after closure completion (Gubisch) [27].

No surgical dissection occurs between the alar cartilages and the overlying skin. However, intercrural and interdomal sutures can be placed from below. A columellar strut, utilizing a septal cartilage graft, serves as a keystone support initially sutured between the cartilages below the anticipated domal projection. Importantly, in this series, grafts from the resected hump or the alar cartilages were intentionally not used as struts, and neither conchal nor rib grafts were employed.

**Fig. 3:**
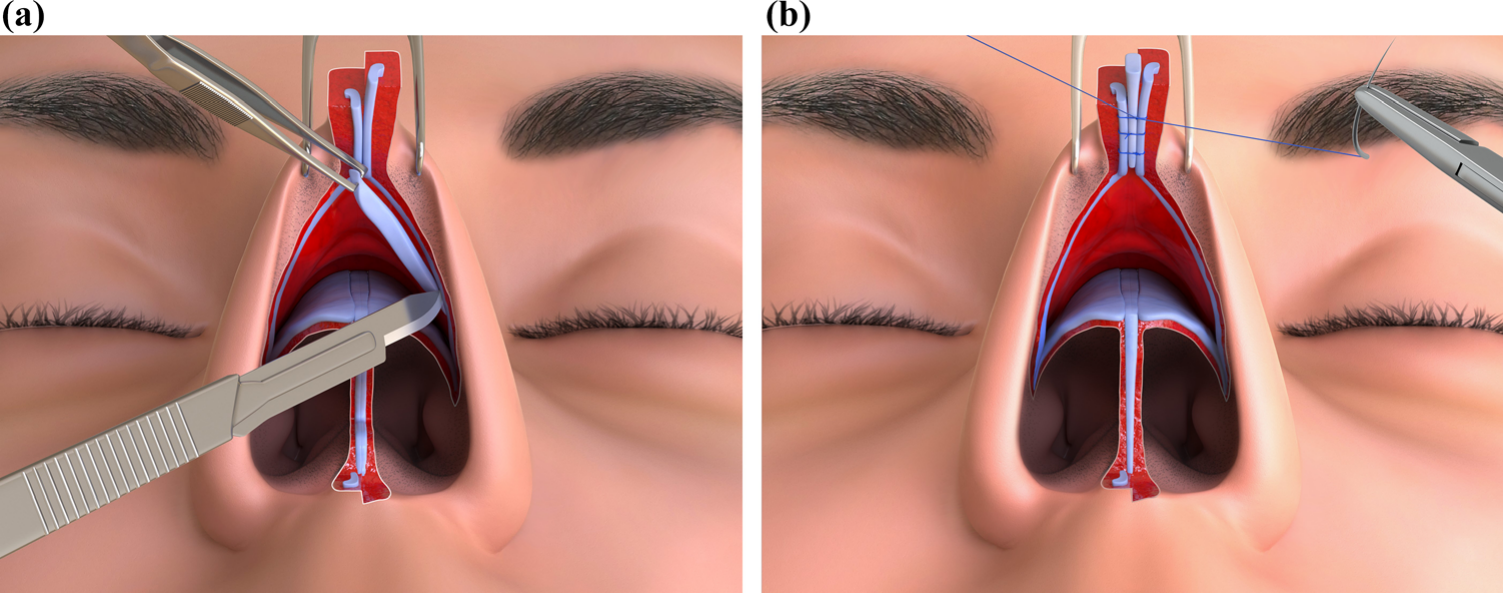
TRICK-TIP rhinoplasty: Preserving the Tip. **a** Without separating the skin from the lower lateral cartilages, a retrograde resection of the cephalic portion can be performed from underneath to achieve tip definition. **b** A strut is sutured in the intercrural space to prevent over projection above the domes. The fixation is first performed in the mobile tip and then secured to the anterior nasal spine and caudal septum

Temporary graft fixation is achieved with a transfixing 25G needle, followed by sutures reinforcing the medial crura. Once the upper end is sutured into the desired position and the final tip height is determined, any length excess of the graft is trimmed. The strut is then directly placed on the anterior nasal spine, where it can be sutured. Additional fixation stitches to the caudal septum using figure-of-eight sutures may also be added (Fig. 3).

Wide dissection enables effective skin redraping and direct access for inspecting central and lateral osseocartilaginous unions. This ensures correcting height differences between nasal bones and cartilages, preventing step deformity and avoiding the inverted "V" stigma. In cases of excessive intraoperative bleeding or floppy skin, external running sutures like Auersvald’s net may be used for three days [28–30].

**Fig. 4:**
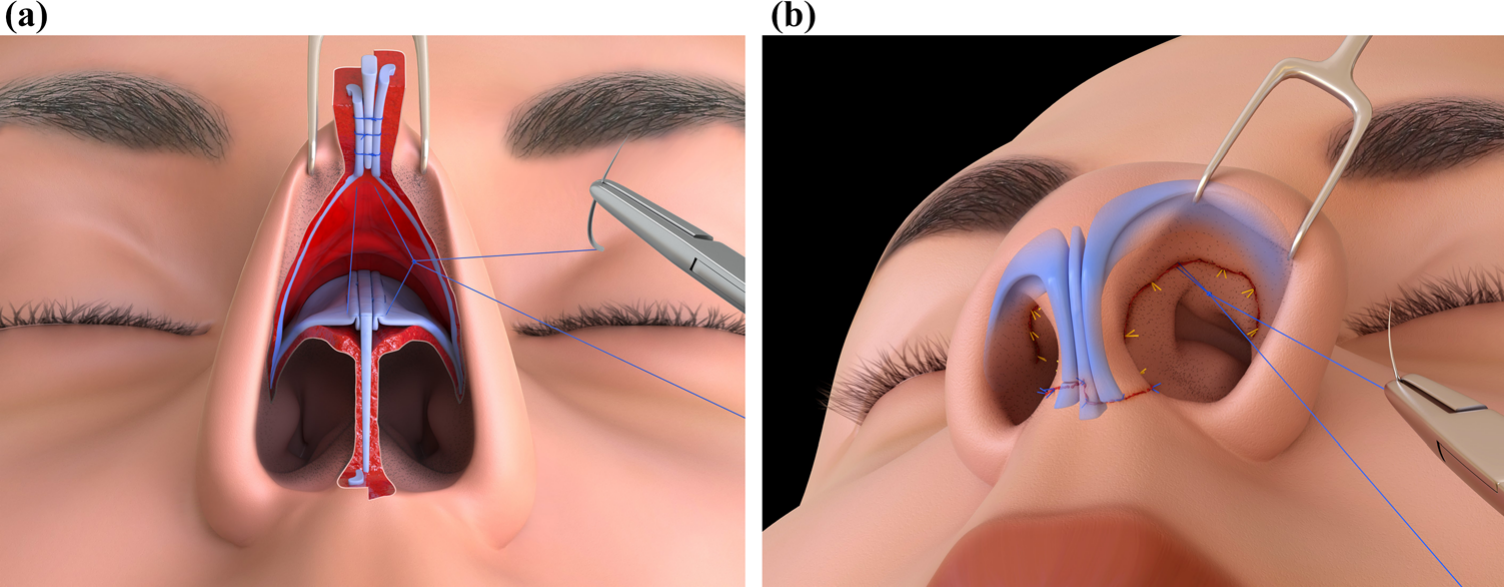
Wound Closure is simplified in TRICK-TIP rhinoplasty. **a** A supratip suture is placed to unite the dermis and cartilaginous vault, closing the dead space at the breaking point. **b** The supratip knot can be tied and buried at the end. It is important to note that the strut has been adjusted and secured based on the desired tip projection

If a Z-plasty is necessary to prevent or correct rim retractions in the vestibular skin, it should be done before closure, utilizing the direct vision provided by the upturned mobile nose. Similarly, placing alar rim struts can be accomplished. Surgical wound closure uses 4-0 USP plain Catgut. The closure sequence starts with the intercartilaginous incision, followed by the membranous septum, and then the external skin with three to four stitches of 5-0 USP polypropylene. Porous paper taping is applied, and a dorsal splint immobilizes. Anterior nasal packing is avoided whenever possible (Fig. 4).

**Fig. 5:**
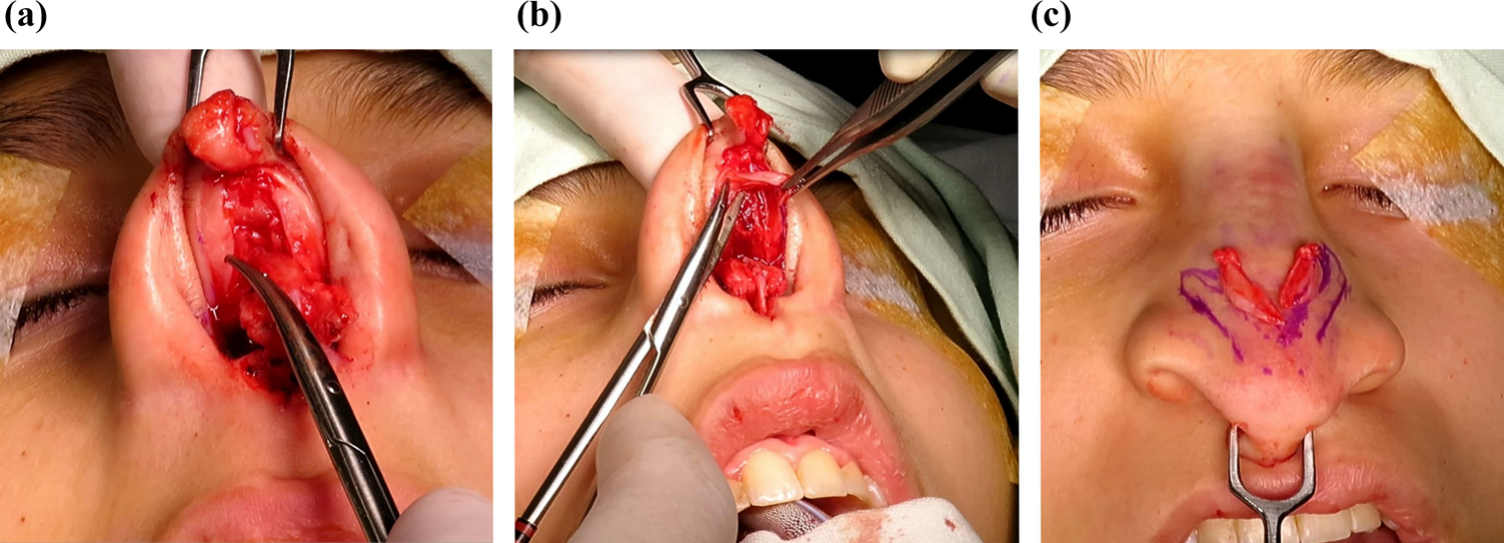
Primary TRICK-TIP rhinoplasty in an 18-year-old female, illustrating the surgery details. **a** Basal view during surgery, showing an uplift of the entire mobile nose as a compound flap. Columellar and intercartilaginous incisions avoid dissection between alar cartilages and tip skin. Littler's scissors point to the cephalic portion of the alar cartilages, marked for modification in a reverse fashion. **b** Trimmed alar cartilages from below, while additional supratip thinning and interdomal resections can be performed in a similar manner. **c** Demonstrative portrayal of the mobile nose turned down, with resected parts superimposed. Purple drawings on the skin indicate remnant alar cartilages for clarity

**Fig. 6:**
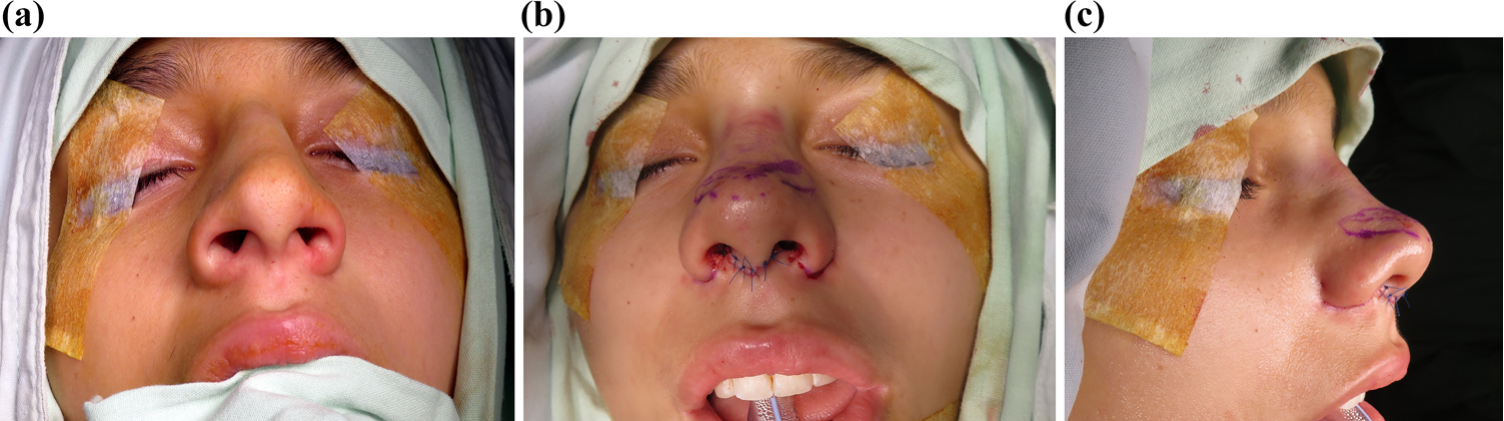
Same patient as in Fig. 5 illustrating the surgery details of alar flaring correction. **a** Basal intraoperative view. **b** Immediately after closure, alar resection was performed, and absorbable suture knots were left inside the nose. **c** Lateral view of the nose demonstrating the lateral extension of alar wedge resection and the absence of external sutures. Also, note the modifications to the dorsum and tip

**Fig. 7:**
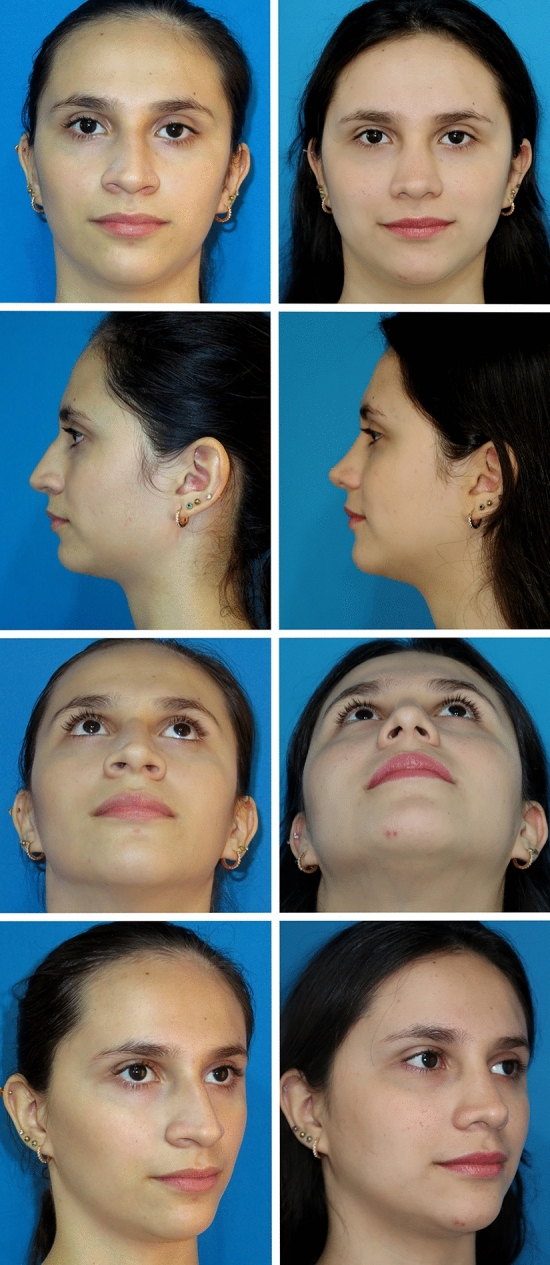
Same patient in Figs. 5 and 6. Primary TRICK-TIP rhinoplasty in an 18-year-old female. Left column: preoperative views. The operative plan encompassed septoplasty, dorsal bony vault resection, chondroplastic modification with spreader flaps of the cartilaginous pyramid, cephalic alar cartilage resection, supratip remodeling, and alar wedge resection. Right: Four months after surgery, the scar is imperceptible, and there is no evidence of crural plates deformity

When necessary, alar flaring correction is performed. After initial demarcation, excision, and hemostasis, a stitch is tied in the nasal sill to achieve the desired nostril width. Transfixing "U" sutures are then placed from the inner nose to the alar crease and back to the nasal vestibule using 4-0 USP plain Catgut. These sutures are left as intranasal knots, typically requiring four to eight stitches. No external sutures are used in the alar crease [31] (Figs. 5, 6, 7).

Postoperative visits are programmed 2 to 5 days after surgery, and then after a week, additional follow-up visits are performed at irregular intervals where postoperative photographs are taken.

Demographics and pertinent clinical and surgical data were extracted from the patient’s charts and databases.

### Evaluation of Results

Results were assessed through patient-reported outcomes and external raters’ evaluation of preoperative and postoperative photographs.

Fifty-three patients reported their outcomes using three validated Spanish FACE-Q™ formularies administered electronically [32–35]. Formularies for scoring “Satisfaction with outcome” (Health-related quality of life), “Satisfaction with nose” and “Satisfaction with nostrils” (Appearance scale), had 5 to 10 evaluable items scoring from 1 (very satisfied) to 4 (very unsatisfied). Raw scores for each set of items were added, producing a total. Using a conversion table based on the Rasch Measurement Theory, scores were converted into a range from 0 (worst) to 100 (best). A fourth FACE-Q™ check list was completed to describe adverse effects relative to rhinoplasty.

External raters, including experienced plastic surgeons and otorhinolaryngologists, were voluntarily invited to answer questionnaires. These raters were online contacts, blinded to patient identities and study authors. Examiners assessed standardized photographs using predetermined rating scales administered through a Google™ questionnaire. Each questionnaire featured preoperative and postoperative photographs of ten patients; respondents could answer one to 12 questionnaires (10 to 120 patients). The platform did not admit incomplete forms, and a respondent could not repeat a series of patients. Rating scales included a five-point scale for assessing “Overall Rhinoplasty Result” (1 the worst to 5 excellent) and a five-point scale for evaluating “Nostril Deformities,” “Soft Triangle Deformities,” and “Columellar External Scar Deformity or Visibility” (ranging from Not Present to Disfiguring).

Descriptive statistics were conveyed through means and their respective standard deviations (SD), and in applicable cases, through medians and their corresponding interquartile ranges. The study examined correlations between various factors such as gender, primary or secondary surgery, previous nose deviation, alar resection during surgery, use of a columellar strut, and the application of a supratip stitch. The Shapiro-Wilk test checked if the data followed a normal distribution; as it did not, the nonparametric Mann-Whitney U test was used for comparisons.

## Results

**Fig. 8:**
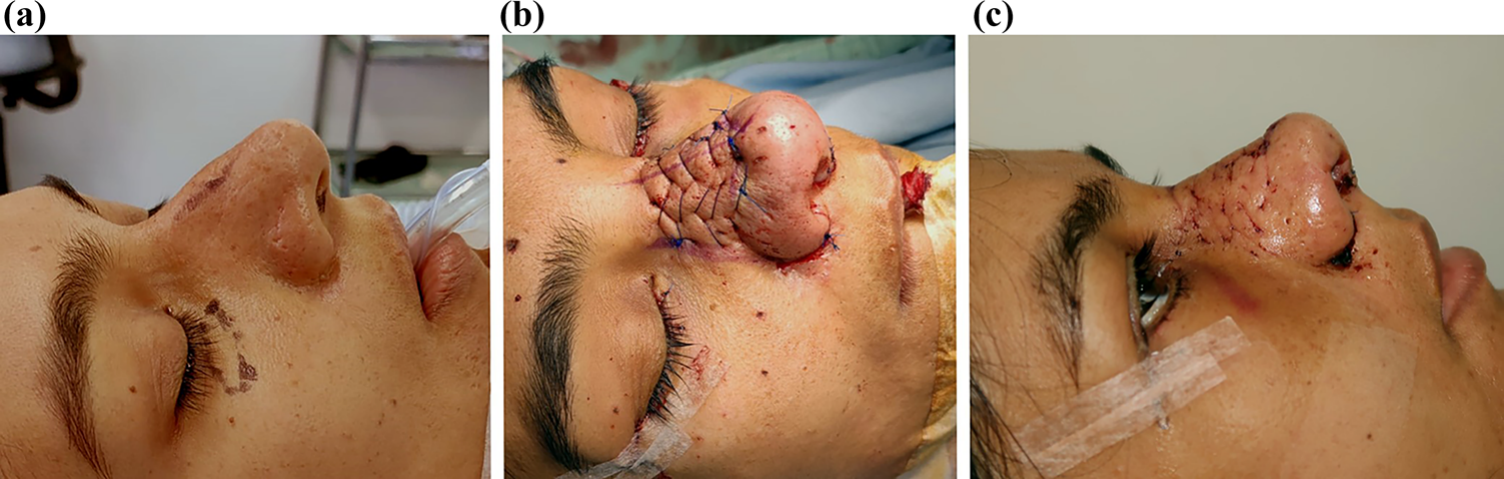
Secondary TRICK-TIP rhinoplasty in a 39-year-old female, intraoperative details. **a** Intraoperative presurgical view: Having undergone a previous reductive rhinoplasty, she presented with concerns of supratip deformity, deviated nose, residual hump, and left alar rim retraction. Simultaneously, a lower blepharoplasty was planned. **b** Intraoperative snapshot post-completion of the surgical plan, including residual hump resection, spreader grafts, vestibular skin z-plasty, left alar rim strut, columellar strut, supratip stitching, and repeated alar resection. Notice the application of a hemostatic net, as described by Auersvald, to accommodate the floppy skin. **c** External sutures are removed 72 hours after surgery

**Fig. 9:**
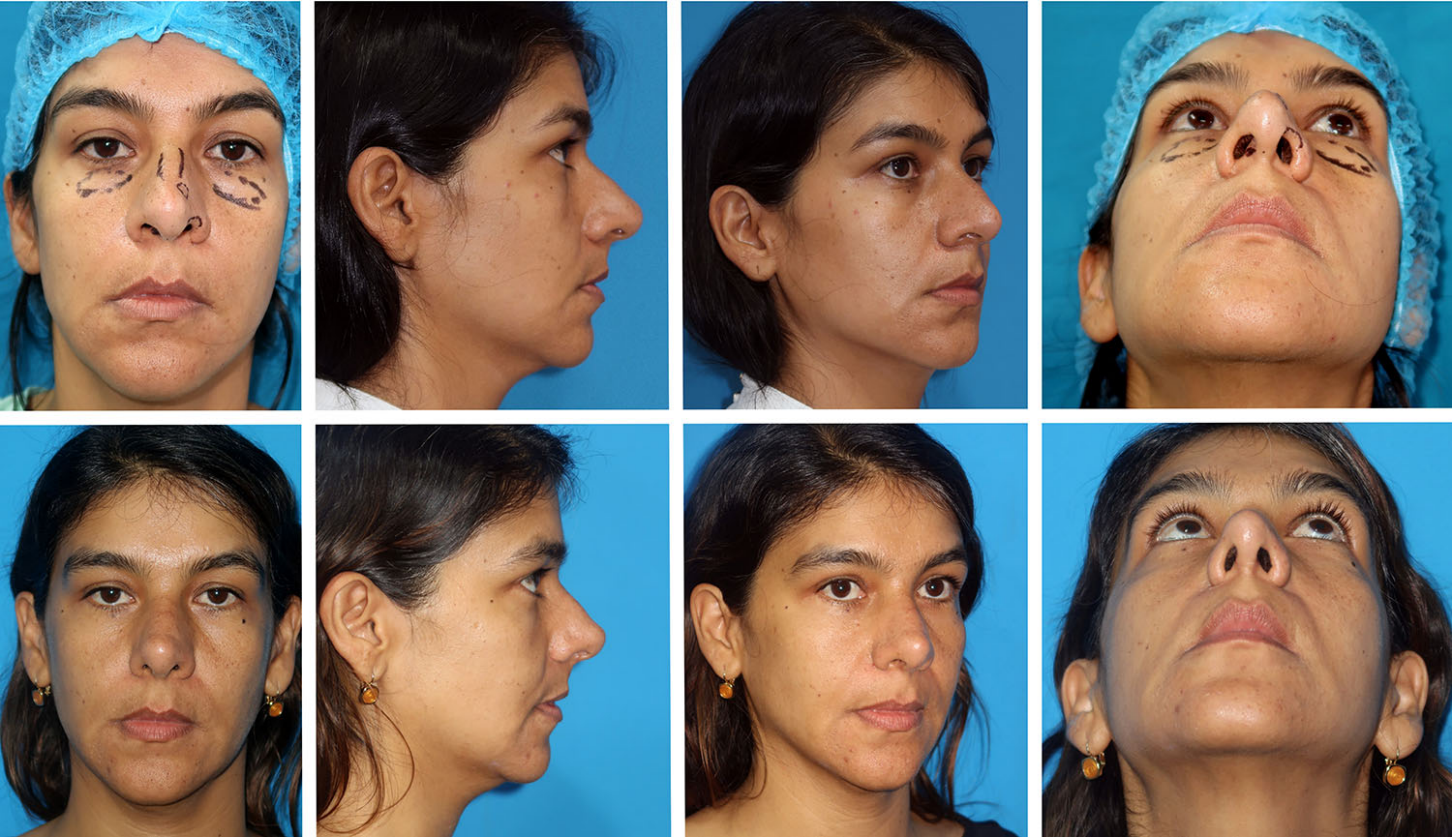
Same patient as in Figure 8. Pre and postoperative views. Top row: Presurgical photos demonstrating residual deformities after a previous rhinoplasty. Besides revisions, a lower blepharoplasty was planned. Bottom row: 6 months after surgery

The study included 120 patients, 86 (71.6%) of whom were females, with an average age of 29.8 (range 17-63). Primary procedures were performed in 106 (88.3%) cases, while 14 (11.7%) were secondary. Thirty-two patients (26.7%) had a main diagnosis of a deviated nose. Rhinoplasty was often combined with other facial (27 cases (22.5%)) or body contour surgeries (31 (25.8%)). The columellar key strut was performed in 52 patients (43.3%), and alar flaring resection was done in 87 (72.5%). The supratip suture was used in 78 (65%). External quilting sutures to the alar creases or extended to other nasal areas were used in 5 cases (4.1%) (Figs. 8 and 9).

### Complications

Skin or columellar necrosis, hematoma, gross asymmetry, wound dehiscences, or infection were not reported; revisions were performed in 3 patients, one of them required two revisions (Table 1).

**Table 1:** TRICK-TIP rhinoplasty patients and surgery characteristics n: 120

Age (years)	Average 29.8 years (range 17–63)
Follow-up (months)	Average 14.1 months (range 3–60)
Females	86 (71.6%)
Males	34 (28.4%)
Primary rhinoplasty	106 (88.3%)
Secondary	14 (11.7%)
Deviated nose diagnosis	32(26.7%)
Use of a key strut	52 (43.3%),
Alar flaring resection	87 (72.5%)
Supratip suture	78 (65.0%)
External quilting sutures	5 (4%)
Associated procedures (Face)	27(22.5%)
Associated procedures (Body)	31 (25.8%)
Complications	Not reported
Revision after TRICK-TIP	4(3.3%)

Patient-reported outcomes indicated high satisfaction with the nose's appearance and the “overall outcome,” achieving a median of 100 out of 100 points. Satisfaction with nostrils received a median of 87 (Table 2).

**Table 2:** FACE-Q™ Patient-reported outcomes after the TRICK-TIP procedure

Patient reported outcome	Number of questions	Median (0 worst to 100 the best
Satisfaction with outcome (Health-related quality of life domain)	6	100
Satisfaction with nose (appearance scale)	10	100
Satisfaction with nostrils (appearance scale)	5	87

Patient reported adverse effects were informed as 'not at all' in 72% of cases and ‘a little’ in 14%. 'Moderately' and 'Extremely' were reported in 7% of cases each, with an average score of 1.5 or lower, indicating a level less than 'a little' on a scale of 1 to 4 (Table 3).

**Table 3:** FACE-Q™ Adverse Effects on the Nose (patient-reported) *n*:53

	1 Not at All	2 A Little	3 Moderately	4 Extremely	AVERAGE (Points)
Difficulty breathing through your nose?	39 (74%)	6 (11%)	3 (6%)	5 (9%)	**1.5**
Tenderness (e.g., when wearing sunglasses)?	36 (68%)	11 (21%)	3 (6%)	3 (6%)	**1.5**
Is the skin of your nose looking thick or swollen?	45 (85%)	5 (9%)	0 (0%)	3 (6%)	**1.3**
Unnatural appearing bumps or hollows on your nose?	34 (64%)	7 (13%)	8 (15%)	4 (7%)	**1.7**
Average (Patients)	**72%**	**14%**	**7%**	**7%**	**1.5 Pt.**

Bold indicates specific numbers or data points of particular importance or interest

One hundred and two independent raters, including 93 (88.6%) plastic surgeons and 12 (11.4%) otorhinolaryngologists, conducted assessments. They evaluated between 10 and 120 patients each, resulting in a total of 1820 evaluations. The “overall rhinoplasty result” across 120 cases had an average rating of 3.62/5 (SD 0.935). Evaluation of ‘Nostril deformities’ yielded an average rating of 1.84 (SD 0.917), ‘Soft Triangle Deformities’ received an average rating of 1.73 (SD 0.903), and the assessment of ‘Columellar External Scar Deformity or Visibility’ resulted in an average score of 1.35 (SD 0.692) Table 4).

**Table 4:** External rater’s qualification of results and deformities after TRIC-TIP procedure (n: 120 pts., 1820 scores)

	Average score	SD
Overall rhinoplasty result 1—Very poor, 2—Poor, 3—Fair, 4—Good, 5—Excellent	**3.62**	0.935
Nostril deformities 1—Not Present, 2—Mild, 3—Moderate, 4—Severe, 5—Disfiguring	1.84	0.917
Soft triangle deformities 1—Not Present, 2—Mild, 3—Moderate, 4—Severe, 5—Disfiguring	1.73	0.903
Columellar external scar deformity or visibility 1—Not Present, 2—Mild, 3—Moderate, 4—Severe, 5—Disfiguring	1.35	0.692

Bold indicates specific numbers or data points of particular importance or interest

The statistical analysis examined six variables to identify correlations with the overall result qualification and the presence of deformities in the nostrils, soft triangles, or columella. No significant differences were observed between groups when comparing ratings for the three potential deformities. However, significant differences were identified in overall rhinoplasty results for cases categorized as secondary and those involving alar resection. Gender, nose deviation, use of a columellar strut, and supratip stitch did not exhibit differences (Table 5).

**Table 5:** External rater’s average qualifications for “overall result” and correlations with six variables^1,2^

Variable	Groups	*n*	Average rating	*p*
Gender	Females	86	3.69	0.88
	Males	34	3.67	
Type of rhinoplasty	Primary	106	3.71	0.010*
	Secondary	14	3.44	
Nose deviation	Present	32	3.57	0.12
	No deviation	88	3.72	
Alar resection	Present	87	3.62	0.005*
	No resection	33	3.86	
Columellar strut	Present	52	3.65	0.395
	No strut	68	3.71	
Supratip suture	Present	78	3.68	0.985
	No stitch	42	3.69	

^1^No differences between groups for qualifications of three possible deformities. ^2^Mann-Whitney U test was used for comparisons. ^*^p < 0.05

## Discussion

The study affirms the effectiveness of the TRICK-TIP surgical approach, emphasizing tip preservation to enhance patient outcomes, and to streamline the procedure. Combining open and closed techniques yielded positive results in patient satisfaction and external raters’ evaluations. Initially employed for relatively straightforward cases, the indications for the technique have diversified widely. It has been utilized in 88% of primary cases and also in 12% of secondary cases. Additionally, the technique has found application in reconstructive surgeries, such as cleft lip repair and severe trauma sequelae. Notably, these latter cases are specifically excluded from this report.

Given the author's experience in both open and closed rhinoplasty, he intuitively recommends the procedure for a broad range of cases. However, it is crucial to highlight that it is not advisable for cases demanding highly precise tip manipulations or for secondary cases requiring alar cartilage reconstruction. The primary indications encompass surgeries involving extensive dorsal modifications, hump removal, correction of nasal sidewalls, and osseocartilaginous junction, particularly when grafts, spacers, and corrections of asymmetries, lateral wall deformities, and septal deviations are necessary.

Unlike traditional open approaches, the TRICK-TIP stands out for preserving the union between alar cartilages and skin, maintaining the integrity of the columella and tip, preventing distortion of the alar rim and soft triangles. This combined approach addresses drawbacks associated with conventional open techniques, such as extensive dissection and scarring, presenting a promising alternative. Its preservative nature also renders the surgery more "reversible and revisable" due to its nondestructive technique.

Vascular safety is crucial in rhinoplasty, especially with alar resection. The technique ensures vascular integrity by avoiding excessive handling of the columellar skin, crucial for procedures involving alar resection.

A key columellar support was incorporated in 43% of patients based on preoperative criteria, including a lack of tip definition and support. In some cases, these criteria were defined intraoperatively. Approximately three-quarters of patients underwent alar resection to correct alar flaring, guided by preoperative, intraoperative, and subjective patient willingness assessments. Suturing to obliterate the supratip space and adhere the skin to the cartilaginous skeleton at the dorsum-tip junction was performed in 65% of cases. This decision was made intraoperatively based on the behavior of the skin during surgery.

**Fig. 10:**
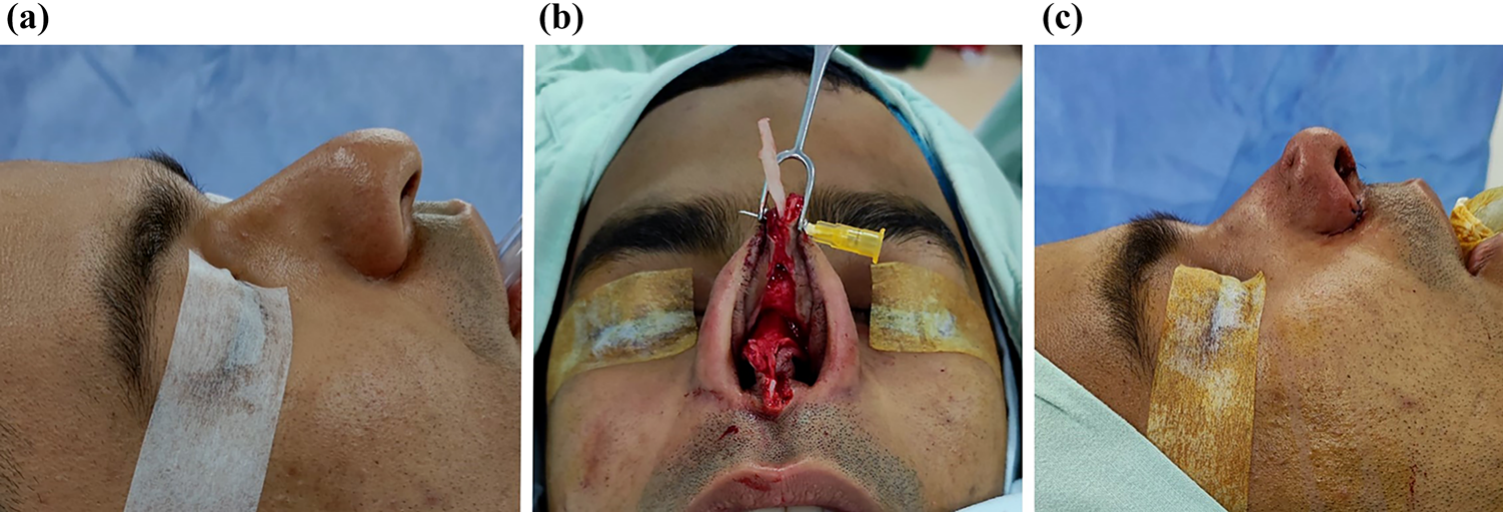
Primary TRICK-TIP rhinoplasty in a 29-year-old male. Use of a key columellar strut. **a** Lateral presurgical view. The patient's main goals included tip projection, narrowing of the tip, and correction of alar flaring. **b** Intraoperative perspective, showcasing a cartilage septal graft placed inlay between the medial crura and temporally secured with a 26G needle. Full fixation is achieved with sutures. **c** Immediate on-table result after TRICK-TIP. Take note of the remarkable tip projection and definition, achieved without the need for alar cartilage dissection. Also, observe a pit, a result of quilting sutures used to prevent supratip deformity

**Fig. 11:**
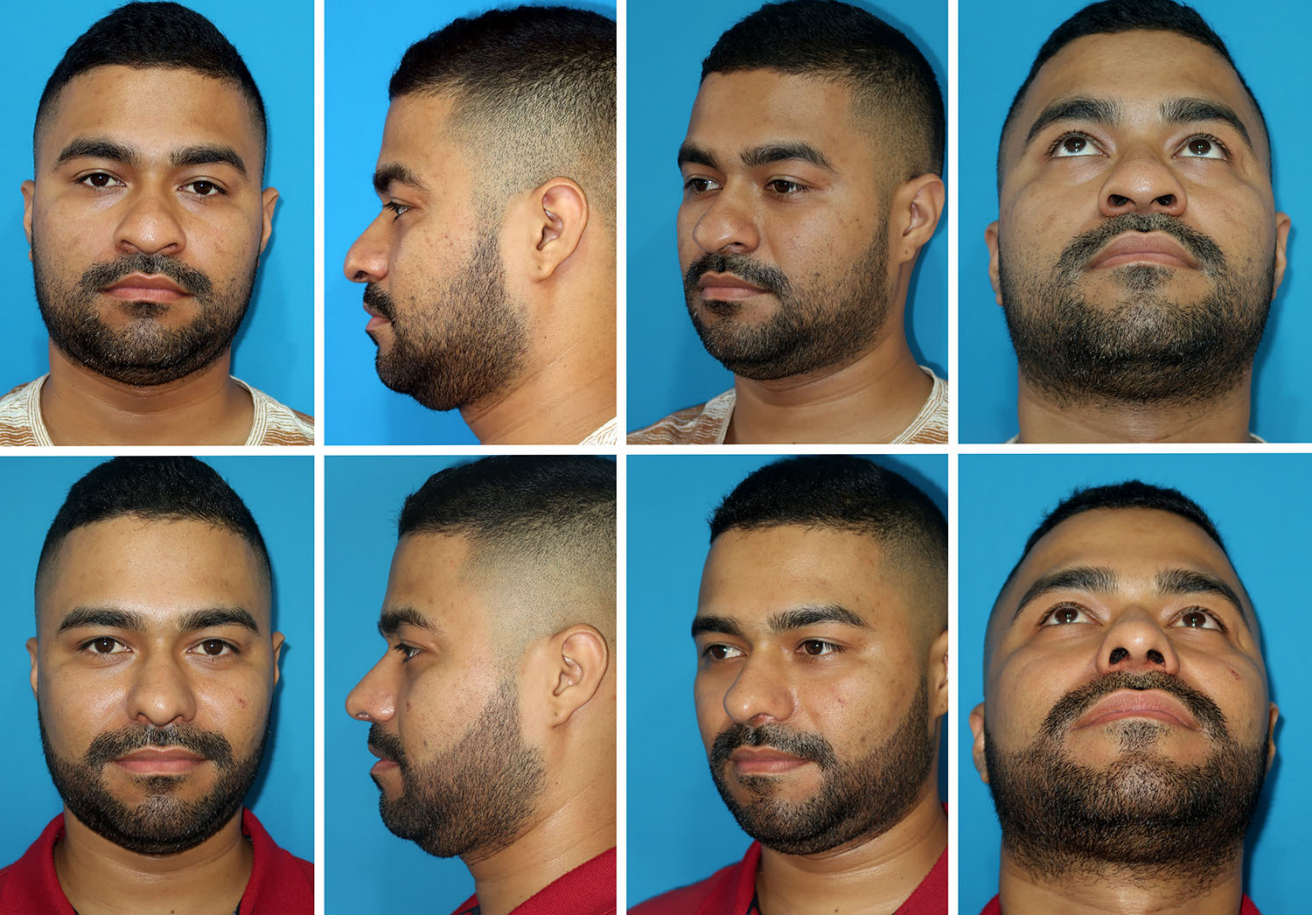
Same patient as in Fig. 10. Top row: preoperative views. Bottom row: seven months post-surgery, demonstrates acceptable results as judged by external evaluators and patient-reported outcomes.

The strategic fixation of the columellar strut, starting in the upper part, facilitates a straightforward maneuver, providing stable tip support. Furthermore, the TRICK-TIP technique enables various tip maneuvers, including retrograde cephalic resection of alar cartilages and interdomal and intercrural sutures (Figs. 10 and 11).

The concept of tip preservation is integral to this approach, as there is no skin separation in the tip-columellar region, maintaining the structural integrity of the support elements and the overlying and internal skin, this preserves the natural contours and characteristics of the nasal tip, resulting in more natural-looking outcomes and reduced risk of tip-related complications. While performing tip modifications from below presents a learning curve and challenges in achieving precise detailed definition, the ease of supporting the tip and direct supratip molding with sutures is a positive aspect. This technique may also offer advantages such as decreased swelling and reduced occurrences of rim or soft triangle deformities due to the absence of skin and cartilage surgical separation.

An analogy can be drawn to Antonov plane in which its pivot nasal elevation allows wide access to the inner part of the aircraft to accommodate large military, agricultural, or complex medical equipment. The acronym "ANTONOV” has been used in podium presentations of the technique to represent the ease and efficiency as follows: Aesthetic, Novel, Technique, Optimizing, Nasoseptal, Openness, and Visibility [36, 37].

Its noteworthy that the term "Novel" should be used cautiously, given historical references to proclaimed new surgeries. However, no antecedent of this specific technique was found in the current medical literature.

A strength of this study lies in its simplicity. Patient-reported outcomes, assessed with a validated scale, and anonymous scoring by more than 100 experienced raters contribute to an unbiased presentation of results and complications. We included a follow-up period averaging 14.1 months (ranging from 3 to 60) in our study to thoroughly examine overall results and the endurance of tip projection, especially concerning the columellar strut. This timeframe covers well-established results while recognizing the possibility of relapse or variations noted at the 3-month mark. This approach contributes to a nuanced understanding of the reported outcomes over time.

Yet, some limitations, such as the descriptive retrospective design, and the need for further generalizability and reproducibility of the technique, should be acknowledged as it matures.

While the current results may not demonstrate a qualitative superiority, the intention is to offer insights into the potential utility of this approach for fellow surgeons. Controlled trials in the future may provide a more comprehensive understanding of its effectiveness when compared to existing techniques.

The assessment of overall rhinoplasty results relied on objective measurements collected from over 100 surgeons, utilizing a scale ranging from 1 (Very Poor) to 5 (Excellent). The average score of 3.62 (SD 0.935) offers an objective measure that aligns with the presented reasonable cases, falling short of outstanding or spectacular results. However, the significance of these objective results becomes apparent when contrasted with an exceptional median score of 100/100 points in the patient reported outcome, FACE-Q™ for 'Satisfaction with outcome' within the Health-related quality of life domain.

The overall outcome, assessed by external evaluators, was analyzed to identify differences based on gender, the presence of nose deviation, alar flaring correction, the use of a columellar strut, the use of supratip sutures, as well as whether the surgery was primary or secondary. Significant differences were found in two out of the six analyzed circumstances. The results were better when alar flaring was corrected, and as expected, outcomes for primary surgeries were better than those for secondary surgeries. The author has transitioned to using more and more this two-in-one approach and recommends surgeons currently employing intercartilaginous endonasal rhinoplasty to consider evolving to sectioning the columellar bridge. This creates a conversion from closed to a wide-open procedure, improving the manipulability of the vault and tip structures.

The TRICK-TIP technique offers a unique approach to rhinoplasty, combining the advantages of open and closed approaches. This innovative surgery widens visibility without columellar and tip dissection of the soft tissue envelope, simplifies the procedure, and maintains favorable outcomes.

Emphasizing vascular safety, diminished risk of complications, the simplicity of the strut fixation, tip preservation, and the ANTONOV metaphor further highlight the distinctive benefits of this approach. Continued research, clinical experience, and surgical education will further validate long-term outcomes, establishing the TRICK-TIP as a valuable addition to the field of rhinoplasty.
